# Supplementary figures and images for: Phosphoregulation of DSB-1 mediates control of meiotic double-strand break activity
Source: eLife. 2022 Jun 27;11:e77956. doi: 10.7554/eLife.77956 (PMC9278955; doi:10.7554/eLife.77956)

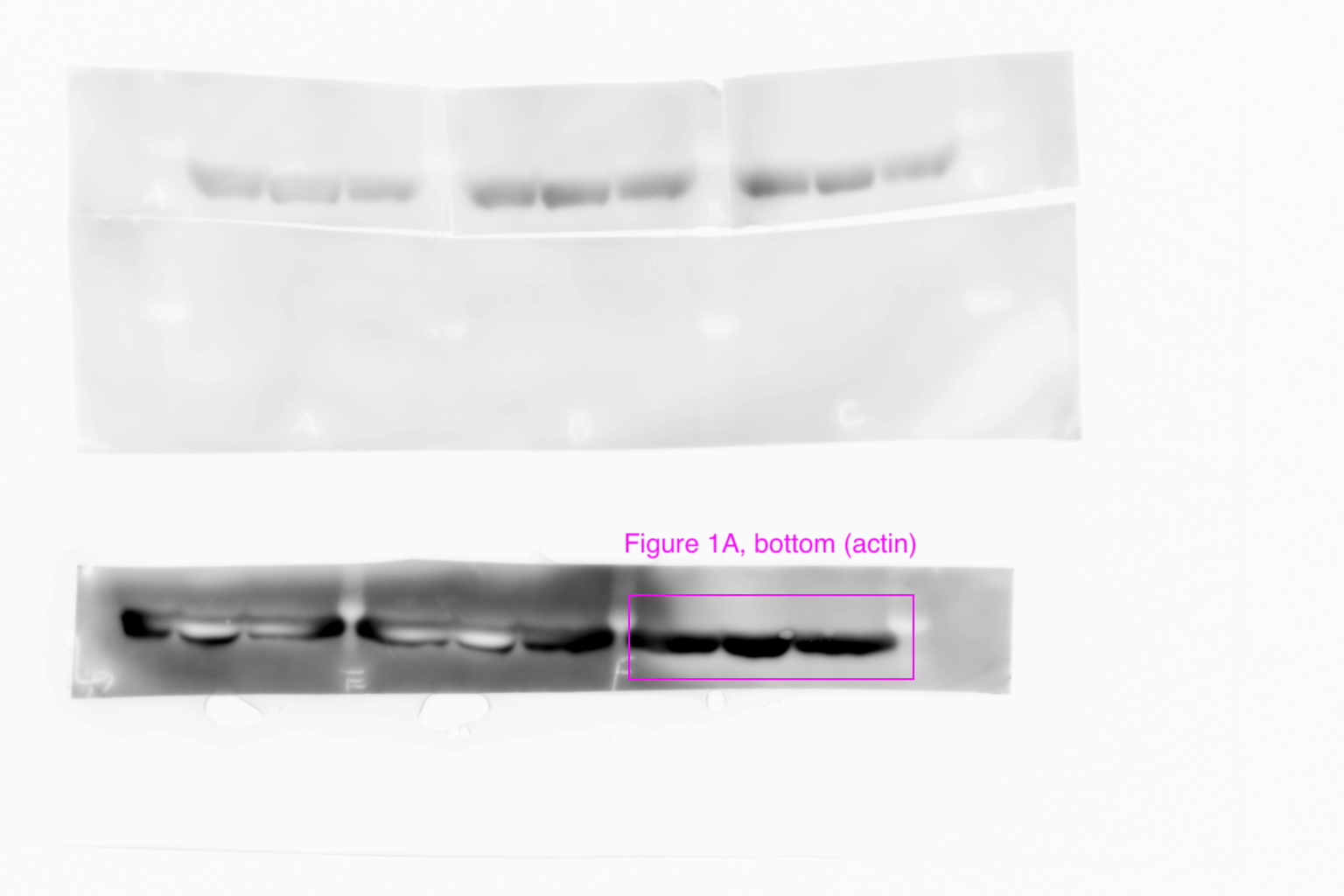

Supplement: Figure 1—source data 2. — Related to Figure 1A. [file elife-77956-fig1-data2.zip › Figure 1–Source Data 2 (actin_labelled).tif]

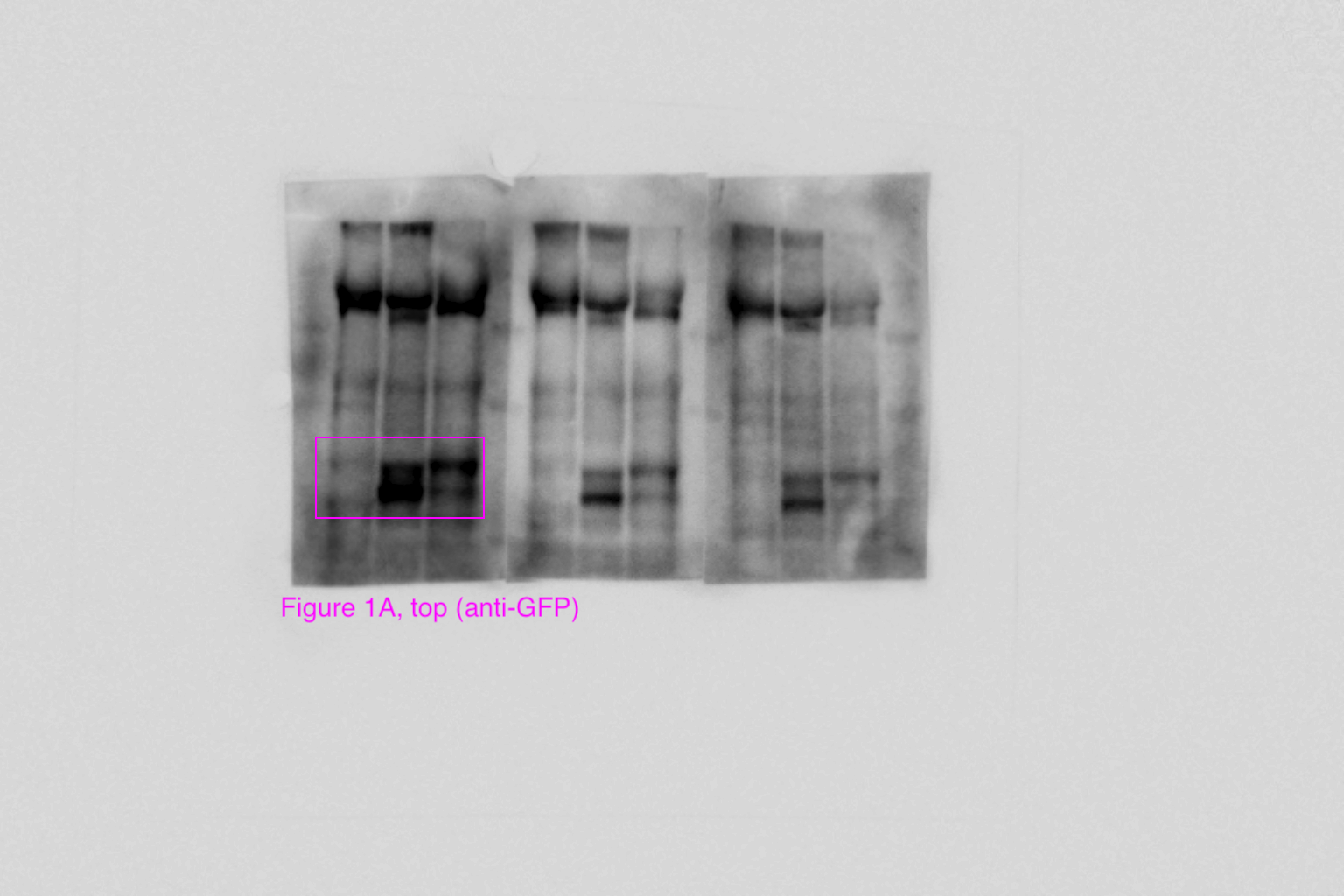

Supplement: Figure 1—source data 2. — Related to Figure 1A. [file elife-77956-fig1-data2.zip › Figure 1–Source Data 2 (GFP_labelled).tif]

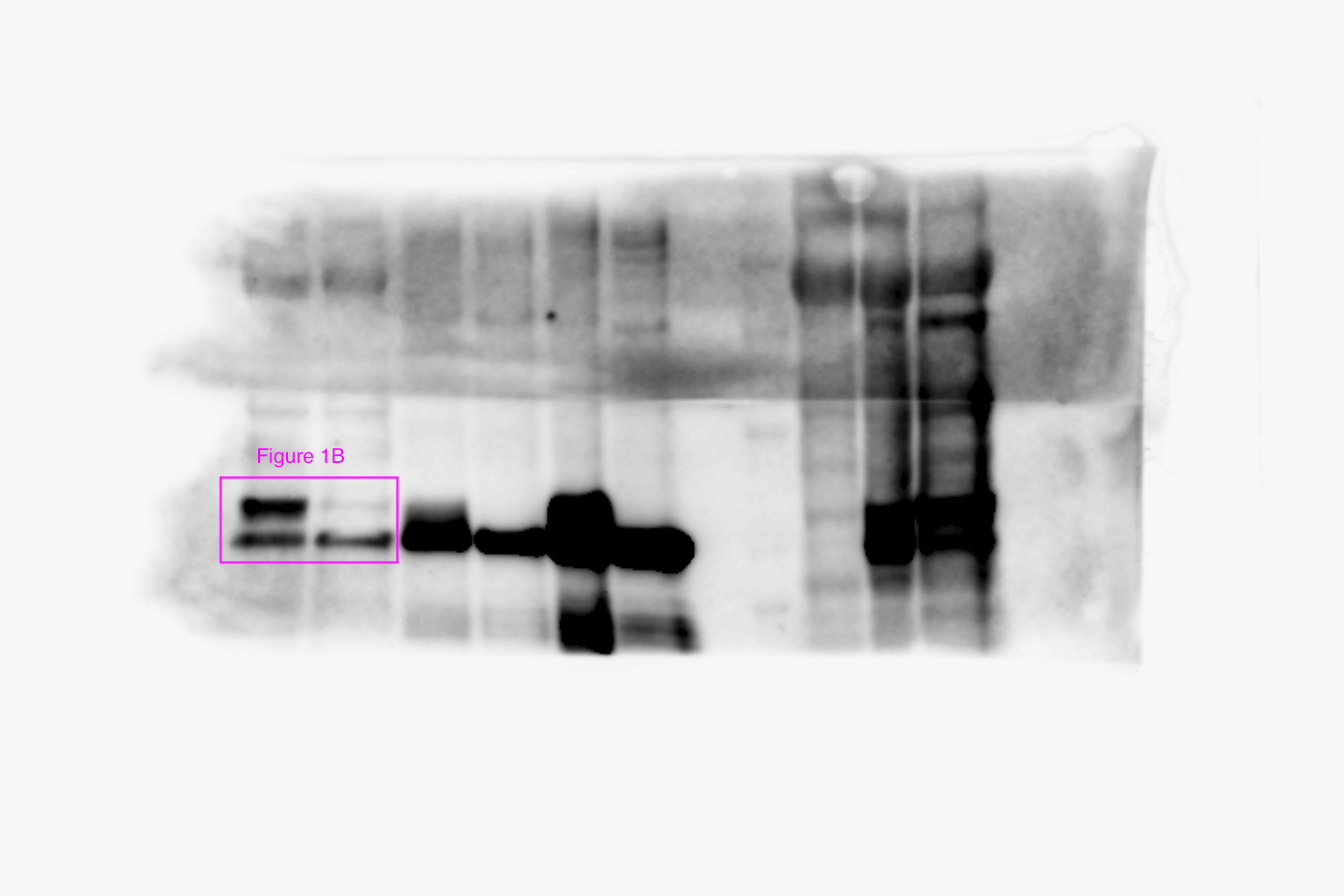

Supplement: Figure 1—source data 3. — Related to Figure 1B. [file elife-77956-fig1-data3.zip › Figure 1–Source Data 3 (labelled).tif]

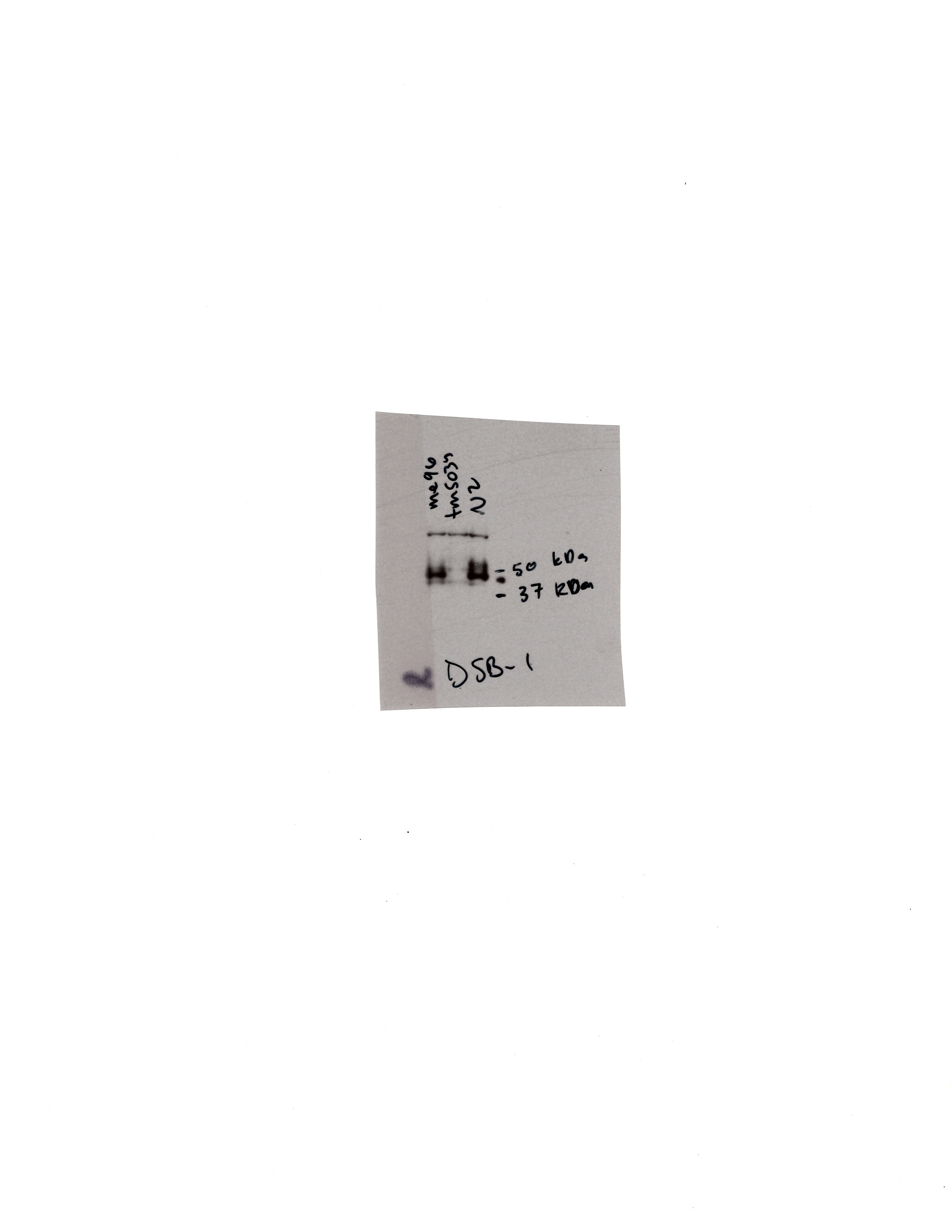

Supplement: Figure 1—source data 4. — Related to Figure 1C. [file elife-77956-fig1-data4.zip › Figure 1–Source Data 4 (unedited).jpg]

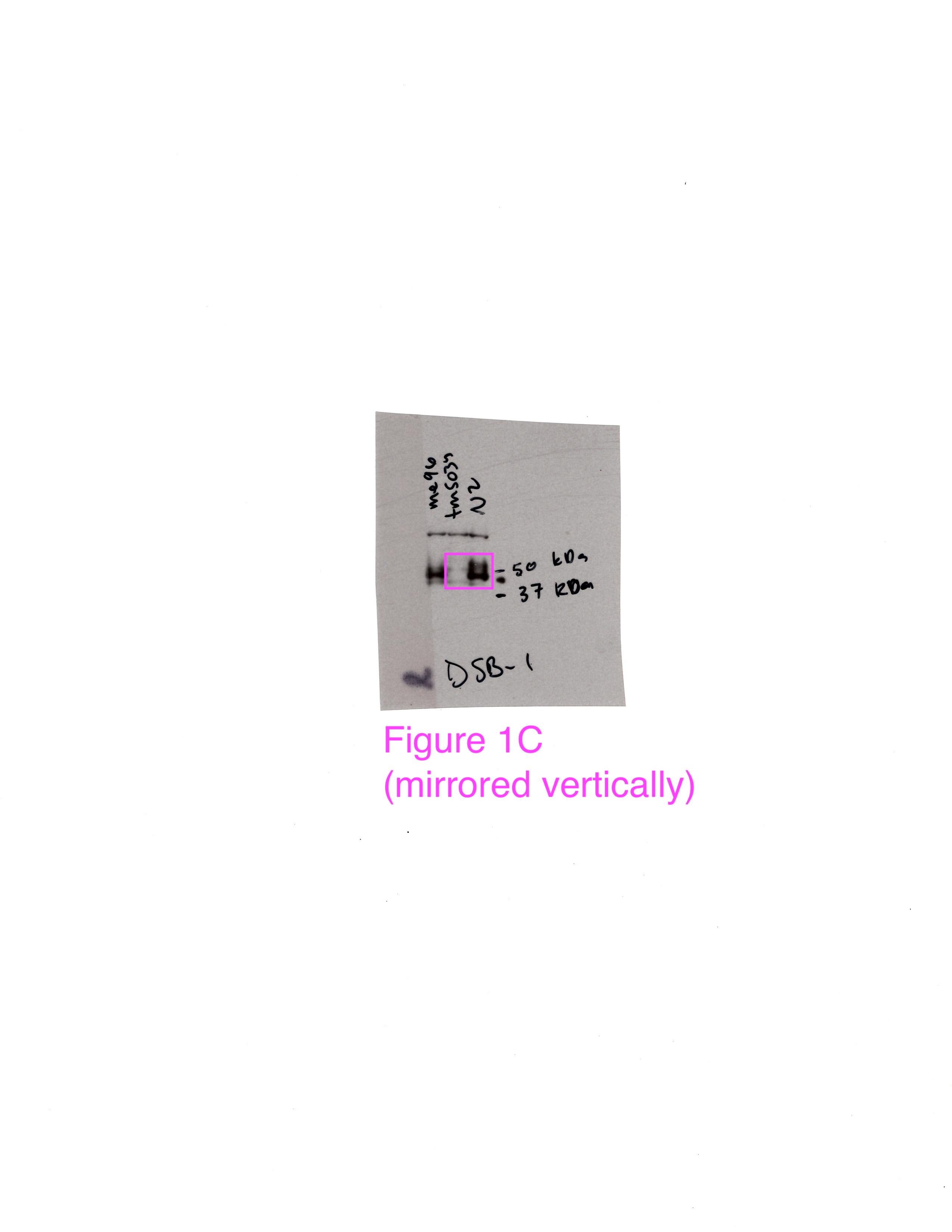

Supplement: Figure 1—source data 4. — Related to Figure 1C. [file elife-77956-fig1-data4.zip › Figure 1–Source Data 4 (labelled).jpg]

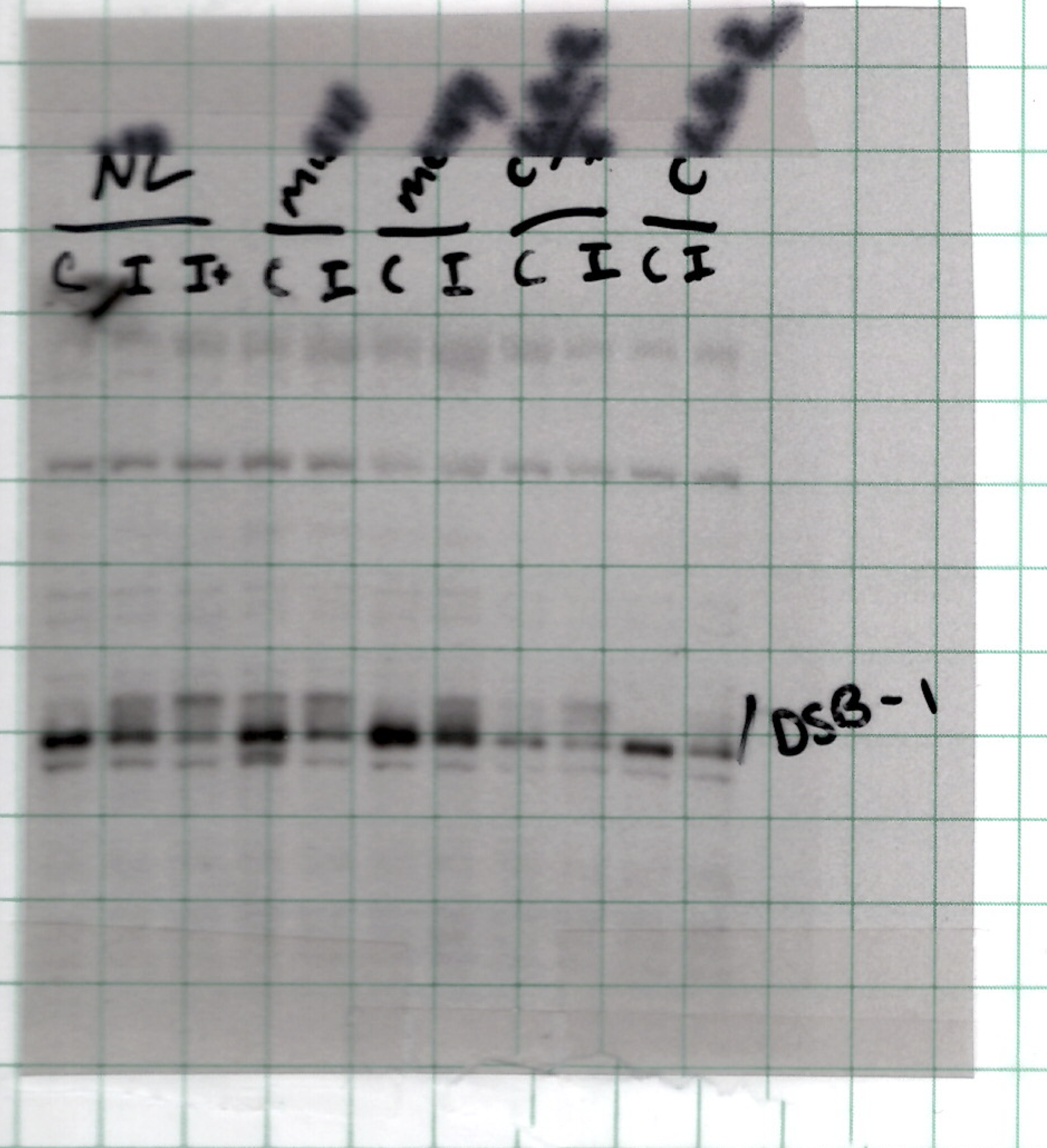

Supplement: Figure 1—source data 5. — Related to Figure 1D. [file elife-77956-fig1-data5.zip › Figure 1–Source Data 5 (unedited).tif]

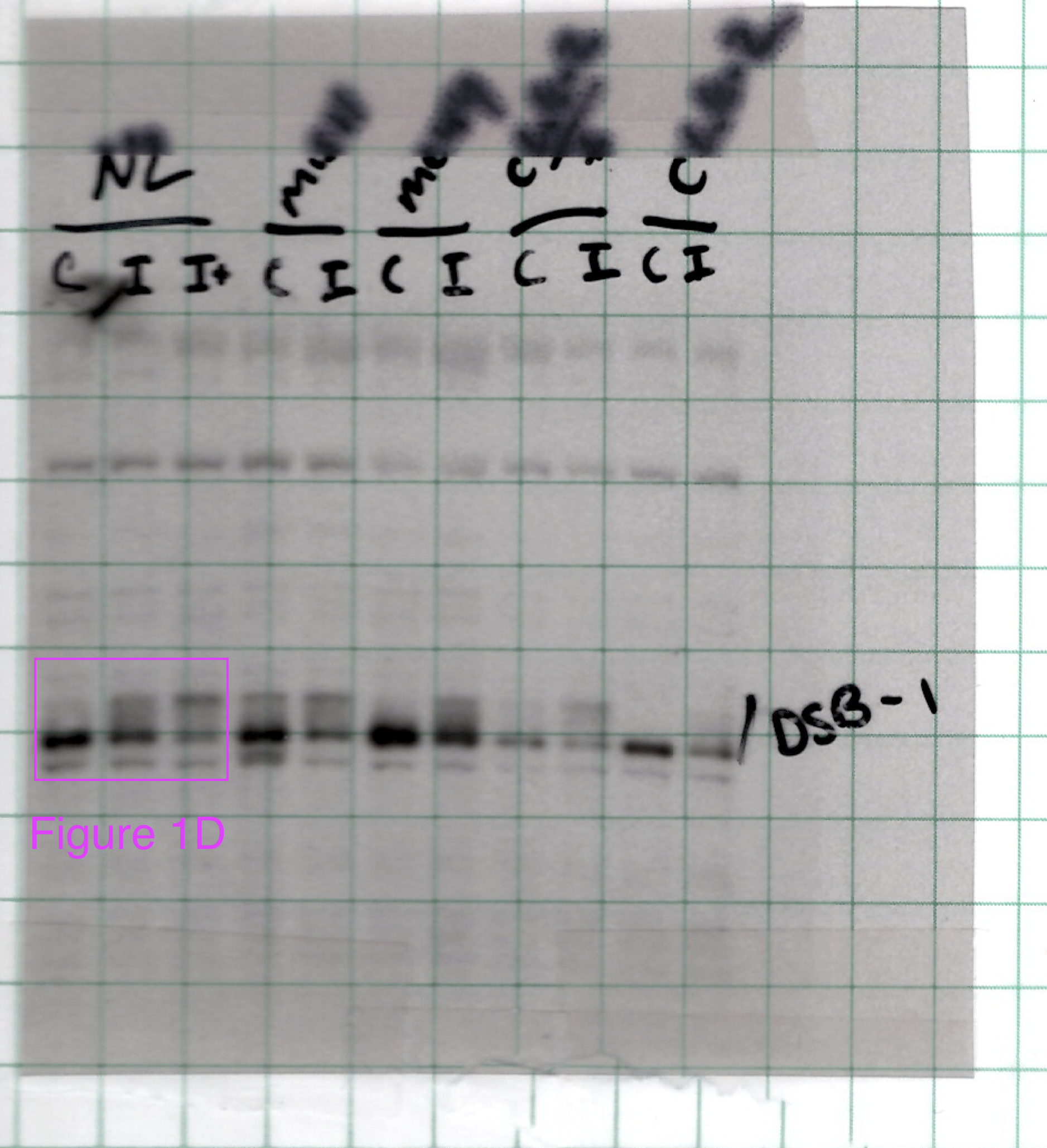

Supplement: Figure 1—source data 5. — Related to Figure 1D. [file elife-77956-fig1-data5.zip › Figure 1–Source Data 5 (labelled).tif]

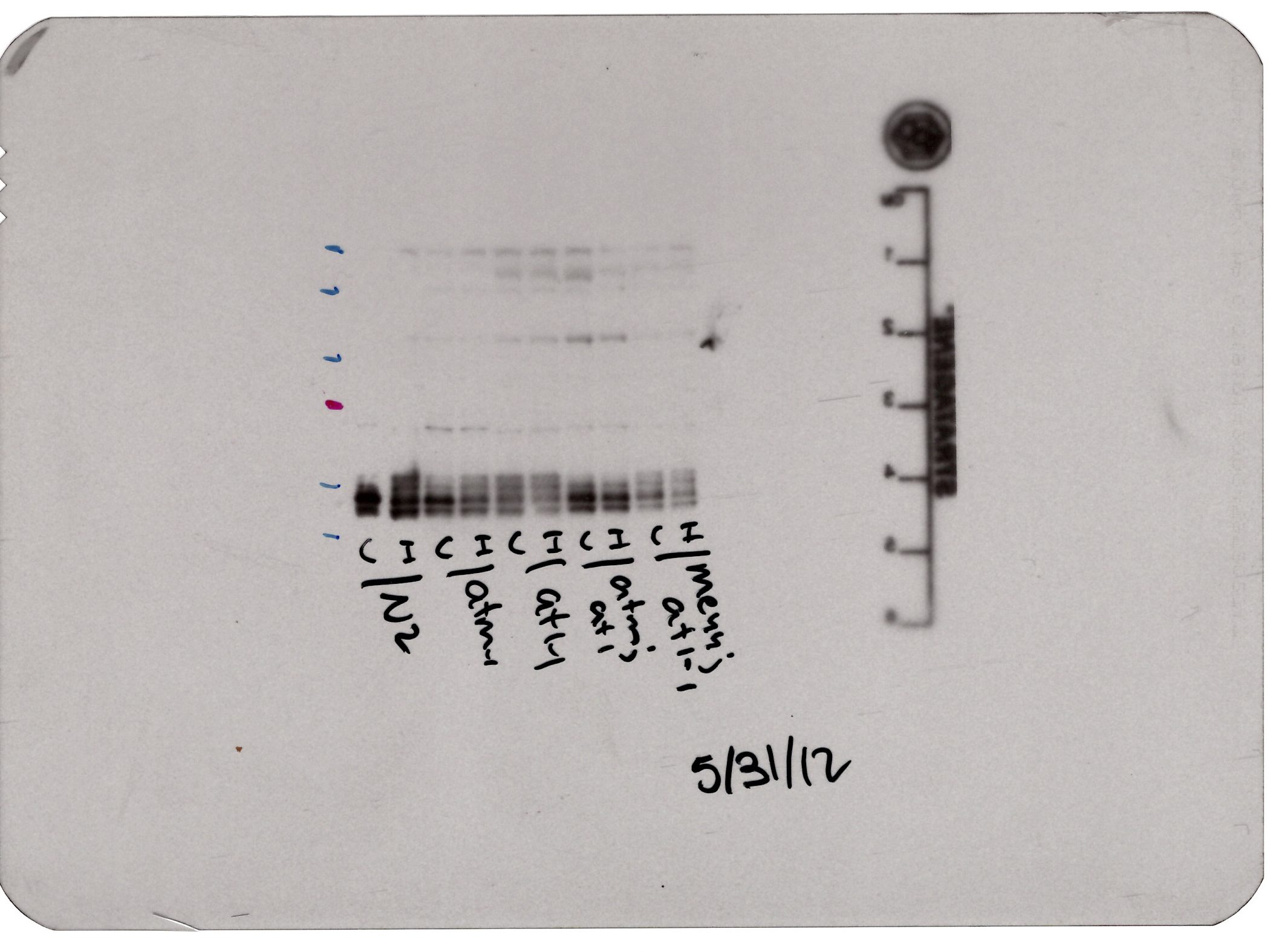

Supplement: Figure 1—source data 6. — Related to Figure 1E. [file elife-77956-fig1-data6.zip › Figure 1–Source Data 6 (unedited).jpg]

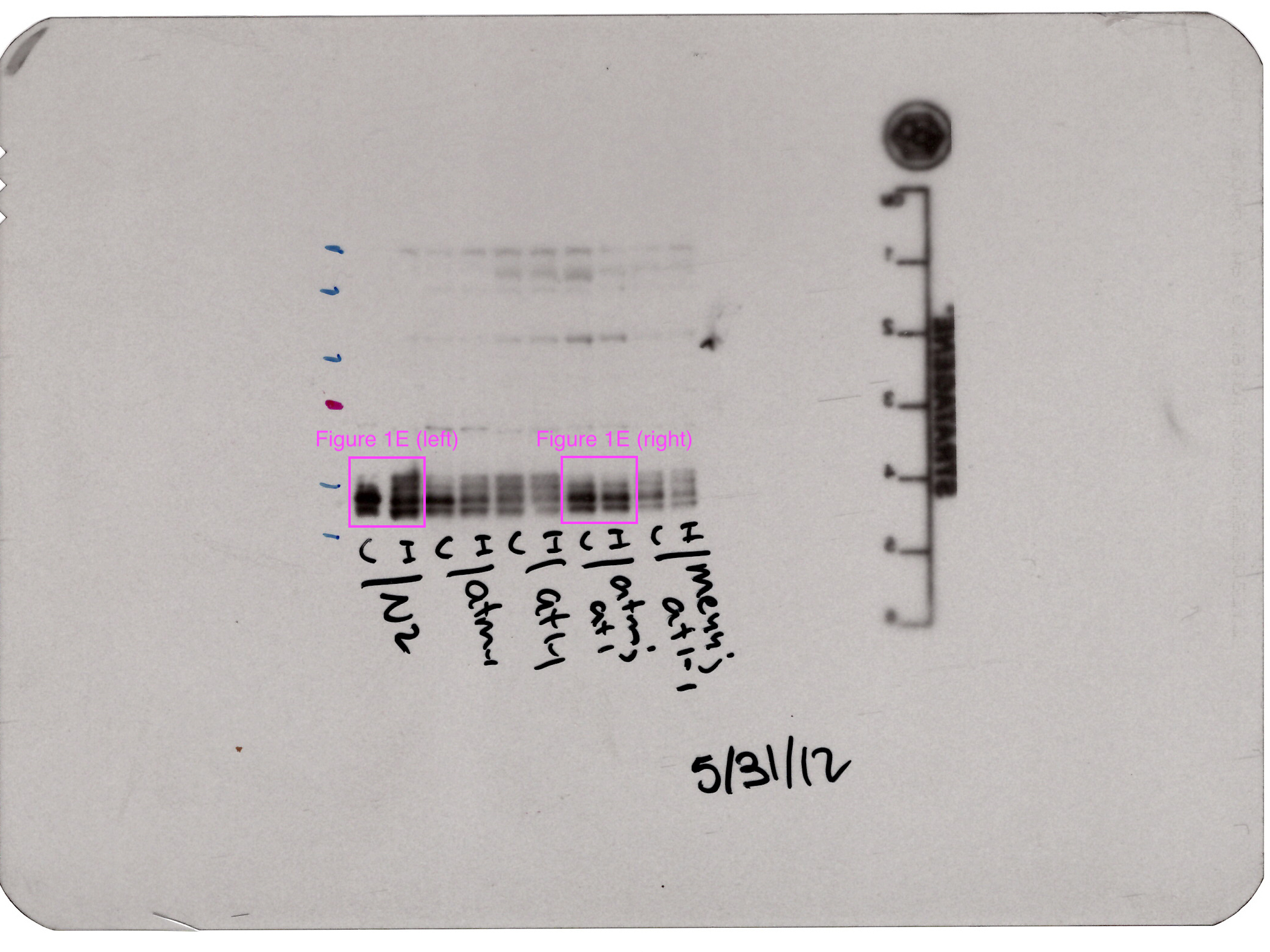

Supplement: Figure 1—source data 6. — Related to Figure 1E. [file elife-77956-fig1-data6.zip › Figure 1–Source Data 6 (labelled).jpg]

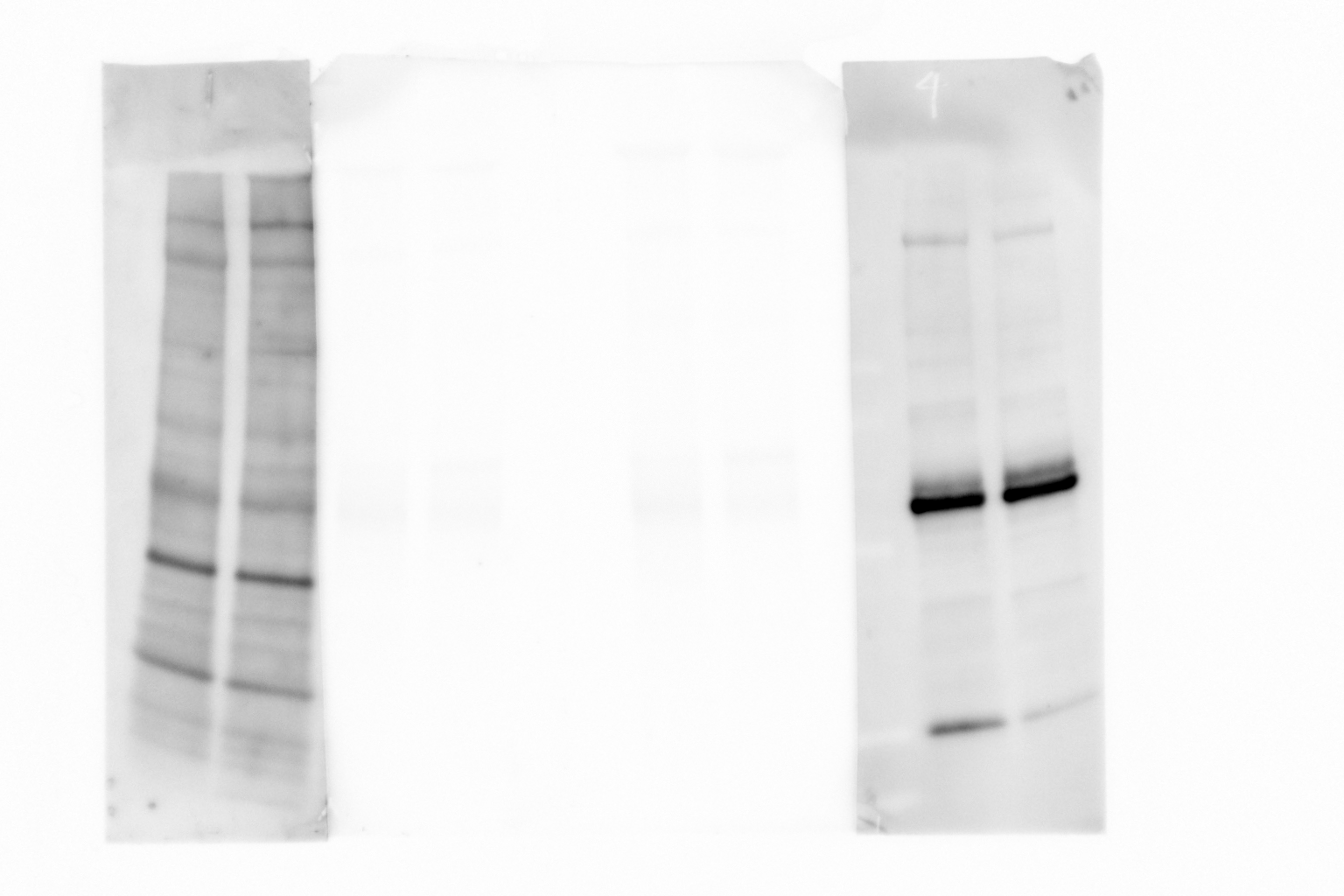

Supplement: Figure 1—figure supplement 1—source data 1. — Related to Figure 1—figure supplement 1B. [file elife-77956-fig1-figsupp1-data1.zip › Figure 1–figure supplement 1–Source Data 1 (actin_unedited).tif]

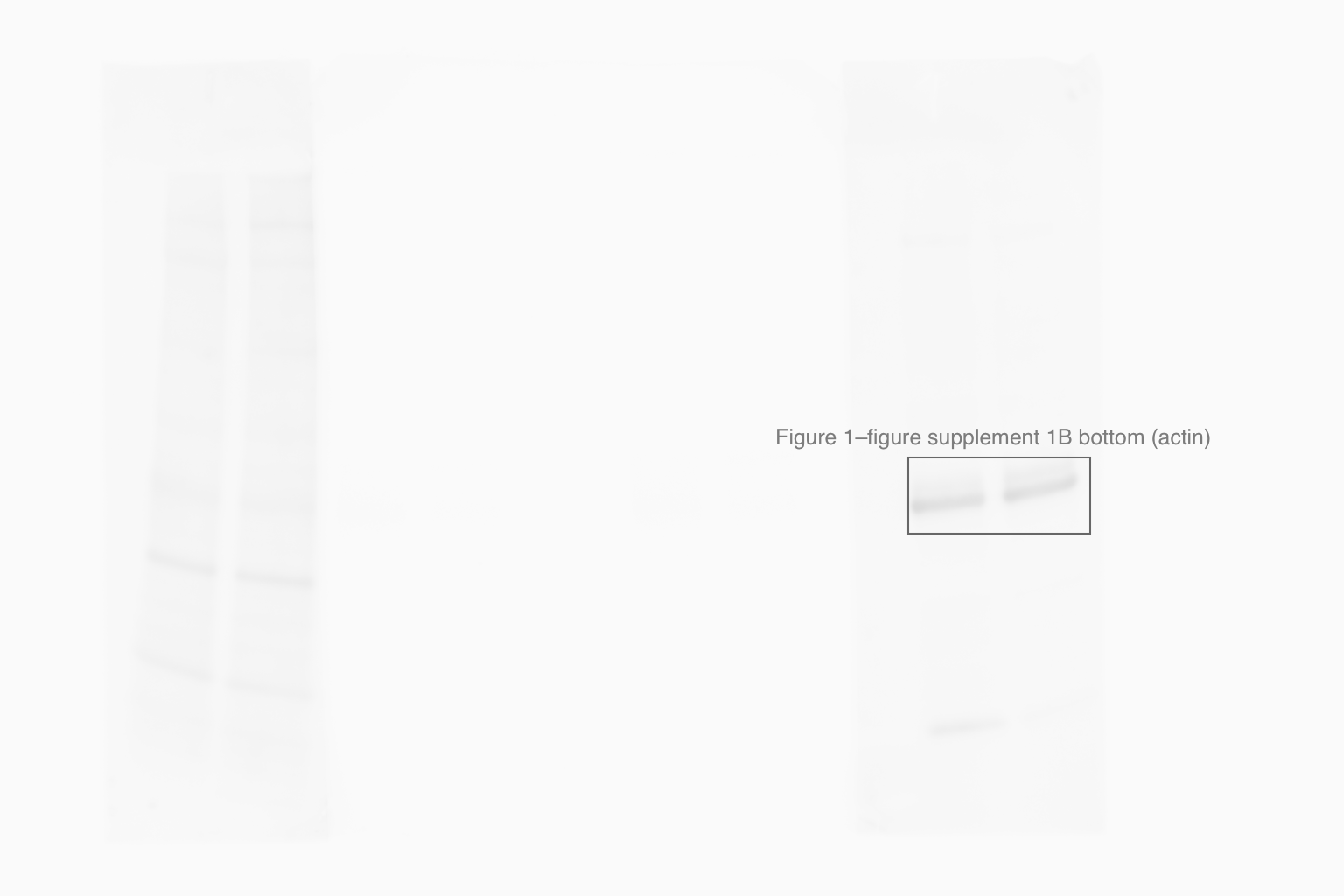

Supplement: Figure 1—figure supplement 1—source data 1. — Related to Figure 1—figure supplement 1B. [file elife-77956-fig1-figsupp1-data1.zip › Figure 1–figure supplement 1–Source Data 1 (actin_labelled).tif]

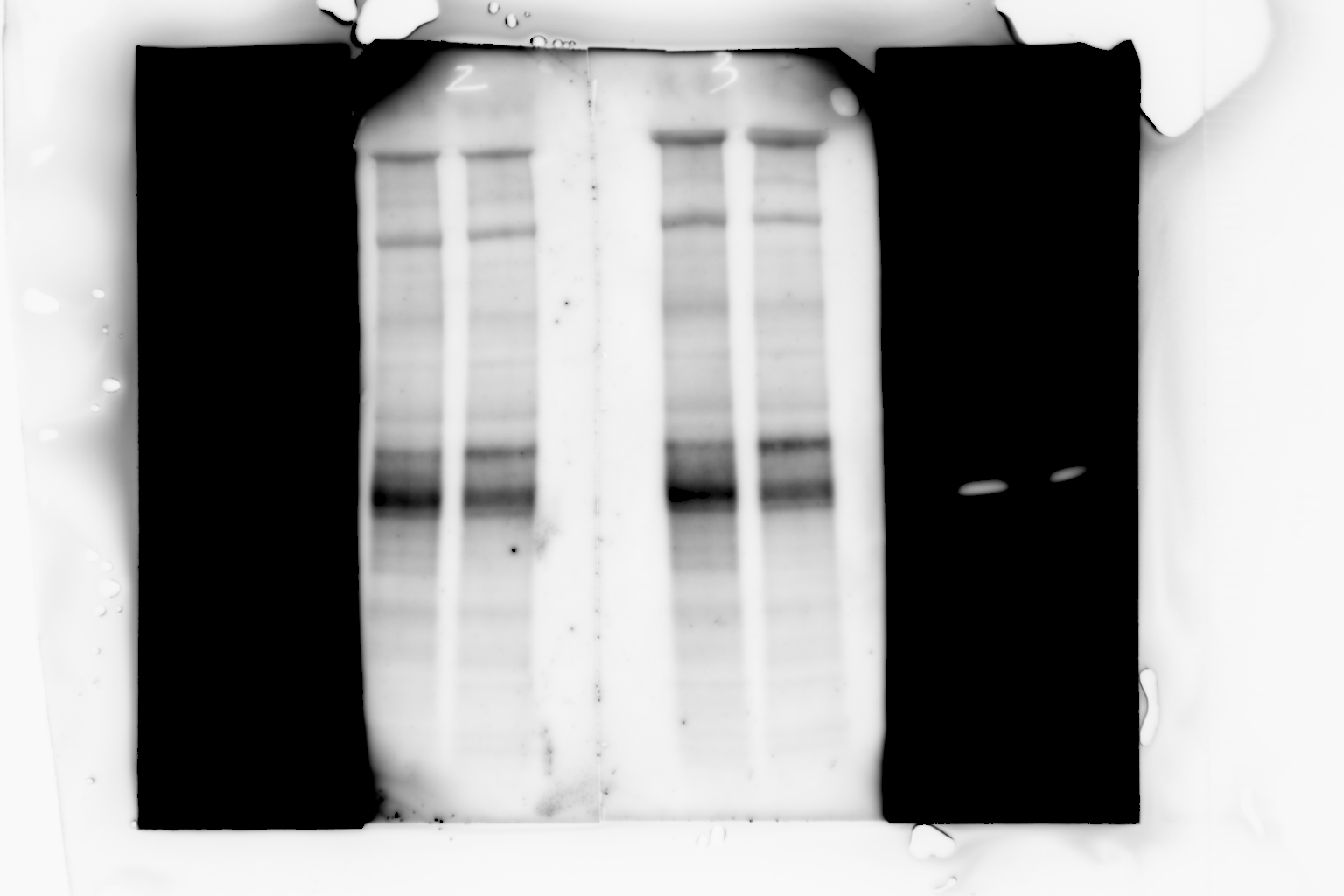

Supplement: Figure 1—figure supplement 1—source data 1. — Related to Figure 1—figure supplement 1B. [file elife-77956-fig1-figsupp1-data1.zip › Figure 1–figure supplement 1–Source Data 1 (DSB-1_unedited).tif]

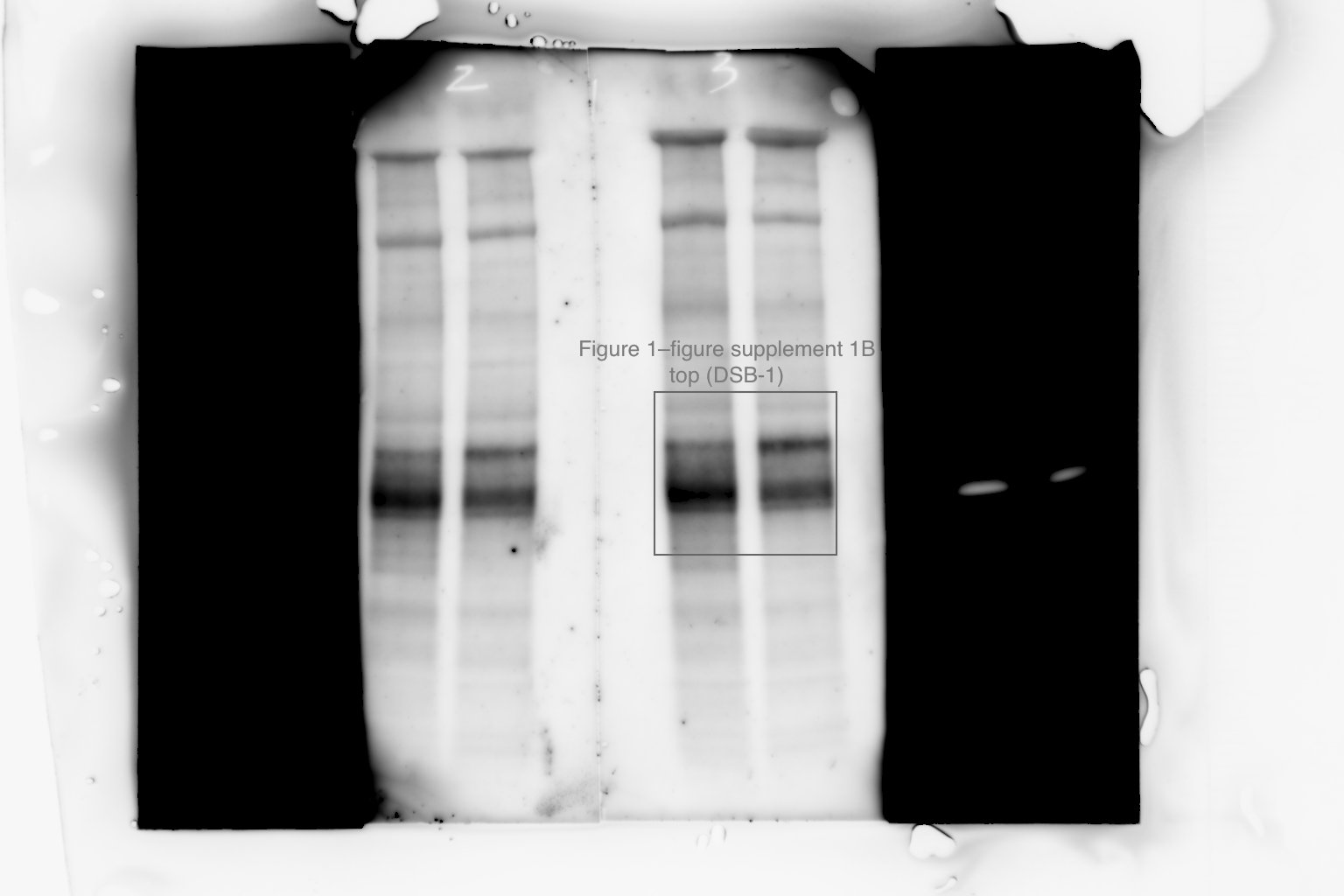

Supplement: Figure 1—figure supplement 1—source data 1. — Related to Figure 1—figure supplement 1B. [file elife-77956-fig1-figsupp1-data1.zip › Figure 1–figure supplement 1–Source Data 1 (DSB-1_labelled).tif]

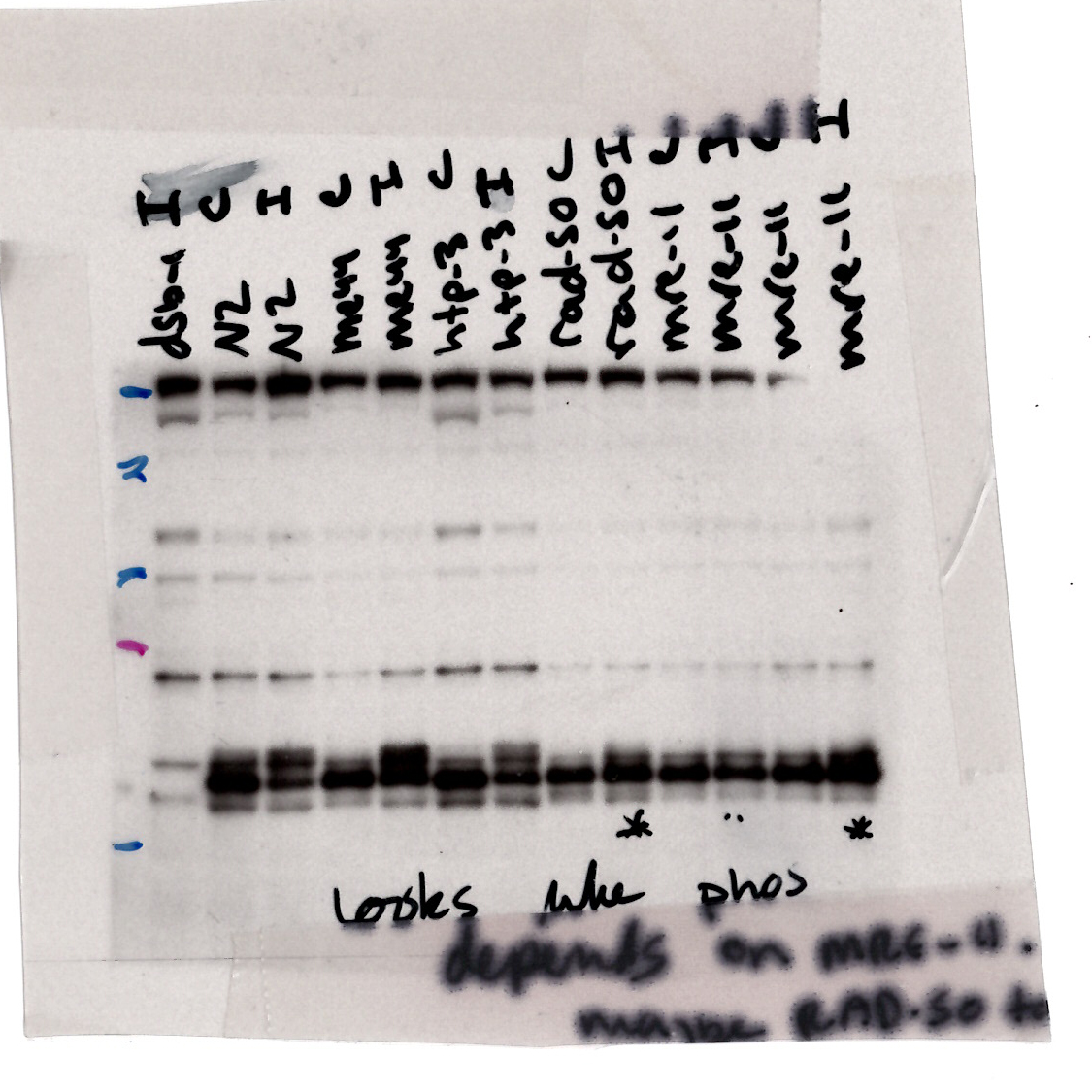

Supplement: Figure 1—figure supplement 1—source data 2. — Related to Figure 1—figure supplement 1C. [file elife-77956-fig1-figsupp1-data2.zip › Figure 1–figure supplement 1–Source Data 2 (unedited).jpg]

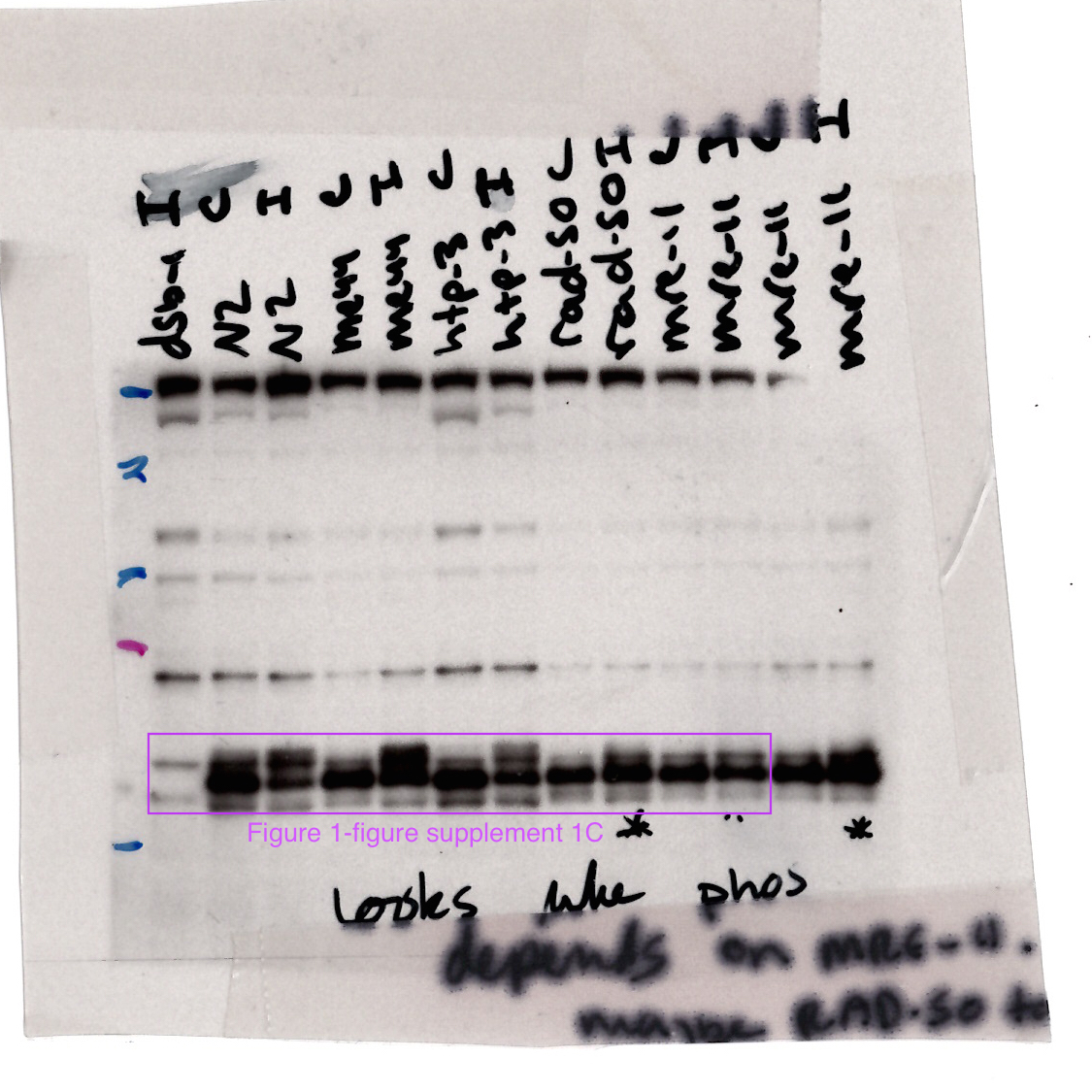

Supplement: Figure 1—figure supplement 1—source data 2. — Related to Figure 1—figure supplement 1C. [file elife-77956-fig1-figsupp1-data2.zip › Figure 1–figure supplement 1–Source Data 2 (labelled).jpg]

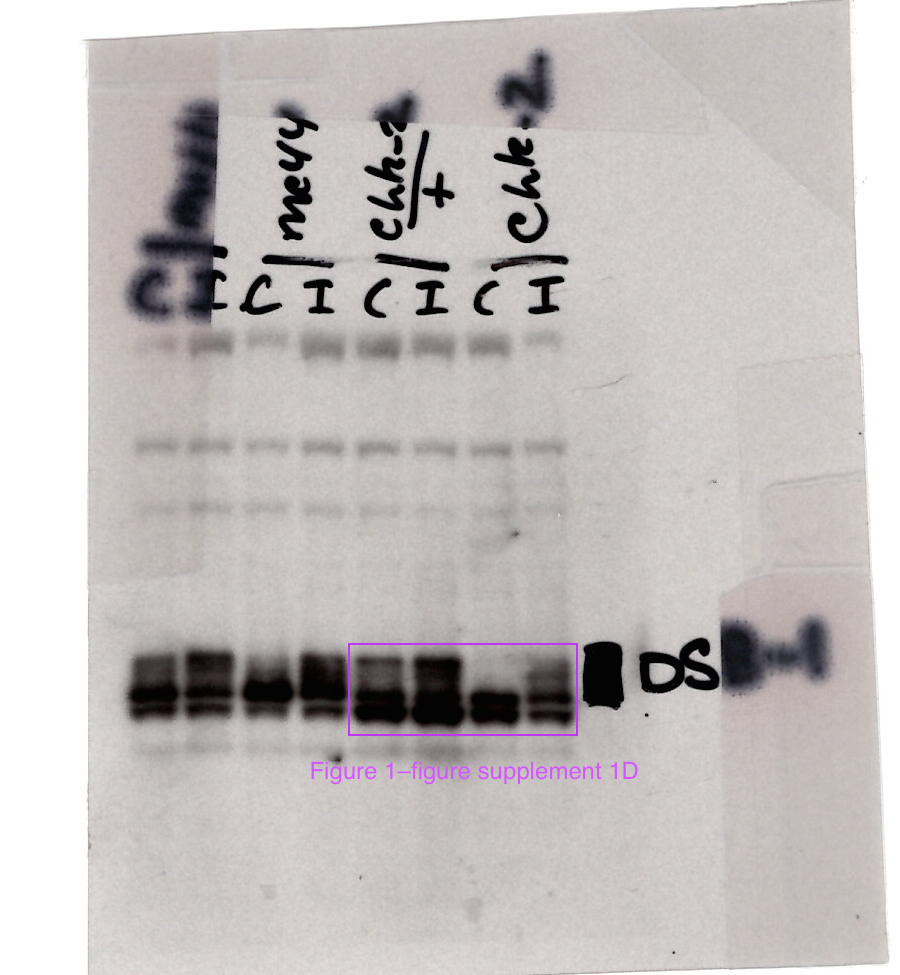

Supplement: Figure 1—figure supplement 1—source data 3. — Related to Figure 1—figure supplement 1D. [file elife-77956-fig1-figsupp1-data3.zip › Figure 1–figure supplement 1–Source Data 3 (labelled).jpg]

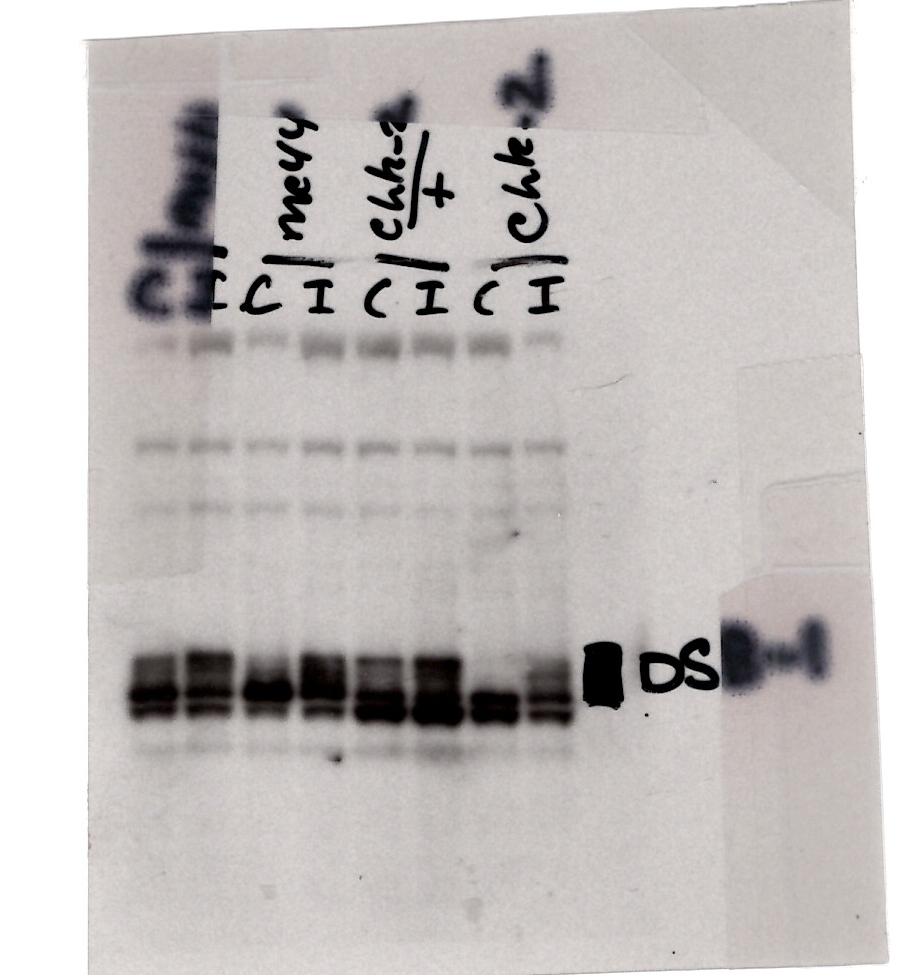

Supplement: Figure 1—figure supplement 1—source data 3. — Related to Figure 1—figure supplement 1D. [file elife-77956-fig1-figsupp1-data3.zip › Figure 1–figure supplement 1–Source Data 3 (unedited).jpg]

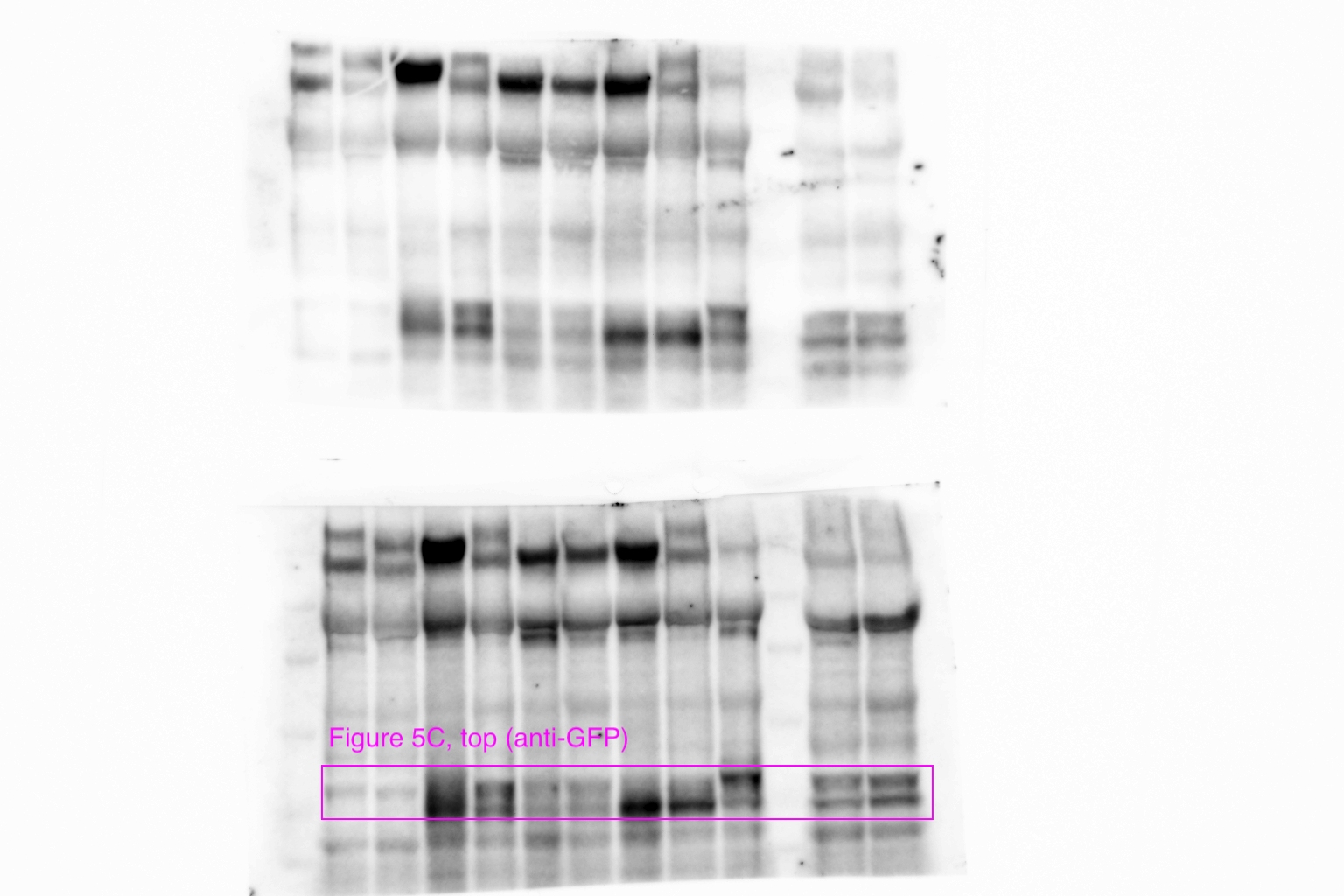

Supplement: Figure 5—source data 3. — Related to Figure 5C. [file elife-77956-fig5-data3.zip › Figure 5–Source Data 3 (GFP_labelled).tif]

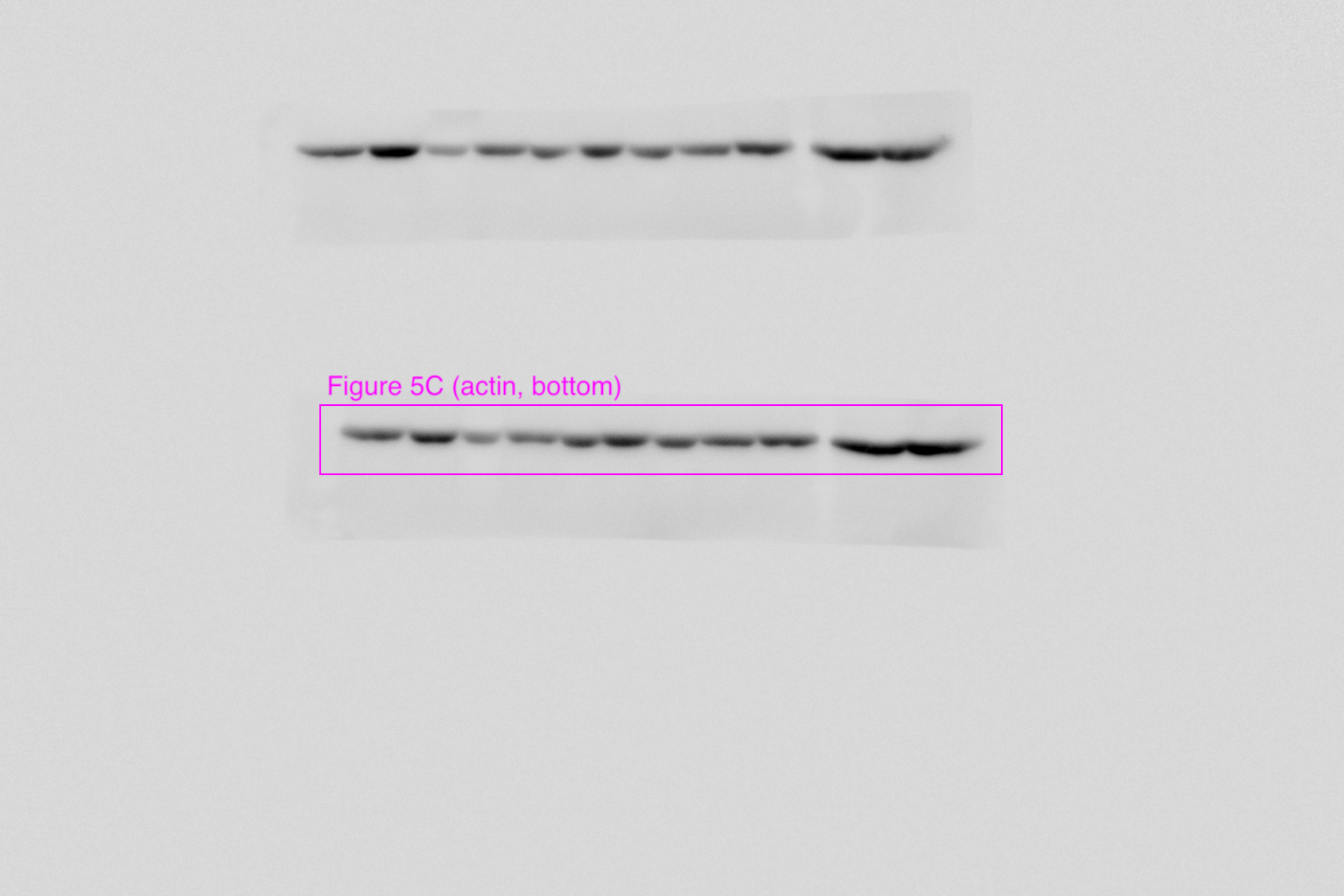

Supplement: Figure 5—source data 3. — Related to Figure 5C. [file elife-77956-fig5-data3.zip › Figure 5–Source Data 3 (actin_labelled)tif.tif]
