# Supplementary material for: Phosphoregulation of DSB-1 mediates control of meiotic double-strand break activity
Source: eLife. 2022 Jun 27;11:e77956. doi: 10.7554/eLife.77956 (PMC9278955; doi:10.7554/eLife.77956)
Supplement: Supplementary file 1. [file elife-77956-supp1.docx]

**Supplementary File 1**

**Strains used in this study**

| **Strains by genotype** | **Source:** | **Strain identifier:** | Additional information |
| --- | --- | --- | --- |
| N2 (wild type) | Brenner, 1974  PMID:4366476 | WB strain ID 00000001 |  |
| *icm97 [gfp-dsb-1](IV)* | This study | PMC575 | Generated in Peter Carlton’s lab, Kyoto University |
| *icm111 [dsb-1(S137A_S143A)](IV)* | This study | PMC537 | Used as the dsb-1(2A) mutant in this paper, generated in Peter Carlton’s lab, Kyoto University |
| *icm112 [dsb-1(S186A)](IV)* | This study | PMC551 | Used as the dsb-1(1A) mutant in this paper, generated in Peter Carlton’s lab, Kyoto University |
| *icm113 [dsb-1(S137A_S143A_S186A)](IV)* | This study | PMC552 | Used as the dsb-1(3A) mutant in this paper, generated in Peter Carlton’s lab, Kyoto University |
| *icm98 [dsb-1(S137A_S143A_S186A_S248A_S255A)](IV)* | This study | PMC569 | Used as the dsb-1(5A) mutant in this paper, generated in Peter Carlton’s lab, Kyoto University |
| *icm114 [gfp-* *dsb-1(S137A_S143A_S186A_S248A_S255A)](IV)* | This study | PMC670 | Used as the gfp-dsb-1(5A) mutant in this paper, generated in Peter Carlton’s lab, Kyoto University |
| *pph-4.1(tm1598)/hT2[bli-4(e937) let-?(q782) qIs48](I;III)* | Sato-Carlton et al., 2014  PMID:25340746 | OC271 |  |
| *dsb-2(me96) (II)* | Rosu et al., 2013  PMID:23950729 | AV477 |  |
| *pph-4.1(tm1598)/hT2[bli-4(e937) let-?(q782) qIs48](I;III); icm97 [gfp-* *dsb-1](IV)* | This study | PMC583 | Used as the pph-4.1; gfp-dsb-1 mutant in this paper, generated in Peter Carlton’s lab, Kyoto University |
| *pph-4.1(tm1598)/hT2[bli-4(e937) let-?(q782) qIs48](I;III); icm112 [dsb-1(S186A)](IV)* | This study | PMC634 | Used as the pph-4.1; dsb-1(1A) mutant in this paper, generated in Peter Carlton’s lab, Kyoto University |
| *pph-4.1(tm1598)/hT2[bli-4(e937) let-?(q782) qIs48](I;III); icm98 [dsb-1(S137A_S143A_S186A_S248A_S255A)](IV)* | This study | PMC594 | Used as the pph-4.1; dsb-1(5A) mutant in this paper, generated in Peter Carlton’s lab, Kyoto University |
| *dsb-2(me96) (II); icm111 [dsb-1(S137A_S143A)](IV)* | This study | PMC630 | Used as the dsb-2; dsb-1(2A) mutant in this paper, generated in Peter Carlton’s lab, Kyoto University |
| *dsb-2(me96) (II); icm112 [dsb-1(S186A)](IV)* | This study | PMC631 | Used as the dsb-2; dsb-1(1A) mutant in this paper, generated in Peter Carlton’s lab, Kyoto University |
| *dsb-2(me96) (II); icm113 [dsb-1(S137A_S143A_S186A)](IV)* | This study | PMC603 | Used as the dsb-2; dsb-1(3A) mutant in this paper, generated in Peter Carlton’s lab, Kyoto University |
| *dsb-2(me96) (II); icm98 [dsb-1(S137A_S143A_S186A_S248A_S255A)](IV)* | This study | PMC580 | Used as the dsb-2; dsb-1(5A) mutant in this paper, generated in Peter Carlton’s lab, Kyoto University |
| dsb-2(me96) (II); *icm114[gfp-* *dsb-1(S137A_S143A_S186A_S248A_S255A)](IV)* | This study | PMC636 | Used as the dsb-2; gfp-dsb-1(5A) mutant in this paper, generated in Peter Carlton’s lab, Kyoto University |
| *atl-1(tm853) V/nT1[unc-?(n754) let-? qIs50] (IV;V).* | [Garcia-Muse and Boulton 2005](about:blank)  PMID:16319925 | DW101 |  |
| *pph-4.1(tm1598)/hT2[bli-4(e937) let-?(q782) qIs48](I;III); atl-1(tm853)/nT1[let-? unc-?(n754)](IV;V)* | This study | PMC253 | Used as the pph-4.1; atl-1/nT1 mutant in this paper, generated in Peter Carlton’s lab, Kyoto University |
| *atm-1(gk186)/hT2; pph-4(tm1598)/hT2[bli-4(e937) let-?(q782) qls48](I;III)* | This study | PMC188 | Used as the atm-1; pph-4.1 mutant in this paper, generated in Peter Carlton’s lab, Kyoto University |
| *atm-1(gk186) rad-54(ok615)/hT2[bli-4(e937) let-?(q782) qls48](I;III)* | This study | PMC193 | Used as the atm-1; rad-54 mutant in this paper, generated in Peter Carlton’s lab, Kyoto University |
| *rad-54&snx-3(ok615) I/hT2[bli-4(e937) let-?(q782) qIs48] (I;III)* | Sato-Carlton et al., 2014  PMID:25340746 | VC531 |  |
| *dsb-1(tm5034) IV/nT1[unc-?(n754) let-?] (IV;V)* | Stamper et al. 2013  PMID:23990794 | CA1105 |  |
| *atm-1(gk186);atl-1(tm853)/nT1[unc-?(n754) let-?] (IV;V)* | This study | CA1079 | Used as the atm-1; atl-1 mutant in this paper, generated in Abby Dernburg’s lab, UC Berkeley |
| *spo-11(me44)/mls11 IV* | Stamper et al. 2013  PMID:23990794 | CA279 |  |
| *htp-3(tm3655) I/hT2[bli-4(e937) let-?(q782) qIs48] (I;III)* | Stamper et al. 2013  PMID:23990794 | CA821 |  |
| *rad-50(ok197) V/nT1[unc-?(n754) let-? qIs50] (IV;V)* | [Hayashi et al. 2007](about:blank)  PMID:17983271 | AV158 |  |
| *mre-11(ok179) V/nT1[unc-?(n754) let-?] (IV;V)* | Stamper et al. 2013  PMID:23990794 | CA1109 |  |
| *chk-2(me64) rol-9(sc148)/+ V* | Stamper et al. 2013  PMID:23990794 | CA1110 |  |
| *spo-11(ie187[spo-11::3xFLAG]), ieSi38[sun-1p::tir1::mRuby::sun-1 3'UTR, Cbr-unc-119(+)] IV; atl-1(ie175[HA::AID::atl-1]) V* | This study | CA1528 | Used as the atl-1 degron allele in this paper, generated in Abby Dernburg’s lab,UC Berkeley |
| *dsb-2( S110A_S116A_S143A_S167A) (II)* | This study | CA1160 | Used as the dsb-2(4A) mutant in this paper, generated in Abby Dernburg’s lab,UC Berkeley |
