# Supplementary material for: Phosphoregulation of DSB-1 mediates control of meiotic double-strand break activity
Source: eLife. 2022 Jun 27;11:e77956. doi: 10.7554/eLife.77956 (PMC9278955; doi:10.7554/eLife.77956)
Supplement: Supplementary file 2. [file elife-77956-supp2.docx]

**Supplementary File 2**

**crRNAs, repair templates and genotyping primers for transgenes generated in this study**

| **Transgenes** | **crRNAs and repair templates** | **Genotyping primers** |
| --- | --- | --- |
| *dsb-1(S137A_S143A)* | crRNA1: 5'-CAACAGTGTCGAGAAACGGA-3' crRNA2: 5'-AATATCGCATGGTTGGGAGA-3' Repair template: 5'-GTTGGTGGAATACGTATGTGATAAGTTTCTTGCCGCGGATTTCTTCACCGAGCCACCAATATCgATgTCGCATGGcTGaGcGAAaGATGTCGGCTGaGcgGAgGAaCCaCCtTTaCGcTTtTCaACggaGTTGTACCACACATctataattcaaaaatgaaacttgaaactattaaaagaataaagaatacCGGTATTTG-3' | forward primer: 5'-GCGTAAAGGTGGTTCCTCCG-3' reverse primer: 5'-GGAGAACGCTGTAAGCCGAG-3' |
| *dsb-1(S186A)* | crRNA1: 5'-CAGTCTACAATCCTTACAAT-3' crRNA2: 5'-TGCTGTGAGTGCTGGCATCC-3' Repair template: 5'-GTGAATCATCGTGGAGAACGCTGTAAGCCGAGCCAATCGATGAACTAACAGTGCTGGCACTCGAgGCTGGaCggTTGTAtGGgTTGTAaACTGGCTGaGcaAAaAAcTCgTTaGCgGTaAGaGCTGGCATaCGaGAACTTTCACCAATGTTGGTGGAATACGTATGTGATAAGTTTCTTGCCGCGGATTTCTTCACCGAG-3' | forward primer: 5'-GTATGCCAGCTCTTACCGCT-3' reverse primer: 5'-AAATGAAGGTGCGTTTGCGG-3' |
| *dsb-1(S137A_S143A_S186A_S248A_S255A)* | crRNA1: 5'-AGGTGCGTTTGCGGGACTGG-3' crRNA2: 5'-GAAAATGACGAACTGTGCGA-3' Repair template: 5'-GCCAAATTCGAACCATTCTTTGCAATTTCCGTGTCCCAGCCCATCtCAttccTCtTCtTTcTCtGGtTTcgCtCAGgtaaattgaaaattaactggaatatcccaccctattaaattaattttcagAGTTCATCACACTCTgCcCAATTGTCtTCCAGTCCCGCAAACGCACCTTCATTTCCGGATTTTCACAGCCCACC-3' | forward primer: 5'-TCCTCTTCTTTCTCTGGTTTCGC-3' reverse primer: 5'-GAACGCATTTGTCCGCAGTT-3' |
| *gfp-dsb-1* | crRNA1: 5'-CTGAATTGCAGACACTCCAG-3' crRNA2: 5'-AATTGCTTATACTTTATGAT-3' Repair template: 5'-caataattttatctgcgatcccaatatgtacagctacgatcccttctcacatacttcctgtgcctgtattttatttattttatttttctttcttcaacgaaccattactatacgggttcccgcttccgaataccgcctttttgactattattccctgcattttcgtgtgaatcattgctcccaagtactcattttgccttccatttggctttaatttattgcagtttgtcgtcataataaactttttatcaatttatcaaaaatttttattttatctattacataaatgcgattaaattttaagttaaaaaaacgagaattagctaaattagatatgcttggtaatttgaatttgaattcgcgcgtaaaagtgatggatgggtctcgccacgaccggcatgcaattttgaattttttcacattttgaggtccaatgaCgtcgaaatttttagacatttttagtaaaaaaaaattatttatgtttttgcagtctcttcagtATGAGTAAAGGAGAAGAATTGTTCACTGGAGTTGTCCCAATCCTCGTCGAGCTCGACGGAGACGTCAACGGACACAAGTTCTCCGTCTCCGGAGAGGGAGAGGGAGACGCCACCTACGGAAAGCTCACCCTCAAGTTCATCTGCACCACCGGAAAGCTCCCAGTCCCATGGCCAACCCTCGTCACCACCTTCTGCTACGGAGTCCAATGCTTCTCCCGTTACCCAGACCACATGAAGCGTCACGACTTCTTCAAGTCCGCCATGCCAGAGGGATACGTCCAAGAGCGTACCATCTTCTTCAAGgtaagtttaaacatatatatactaactactgattatttaaattttcagGACGACGGAAACTACAAGACCCGTGCCGAGGTCAAGTTCGAGGGAGACACCCTCGTCAACCGTATCGAGCTCAAGgtaagtttaaacagttcggtactaactaaccatacatatttaaattttcagGGAATCGACTTCAAGGAGGACGGAAACATCCTCGGACACAAGCTCGAGTACAACTACAACTCCCACAACGTCTACATCATGGCCGACAAGCAAAAGAACGGAATCAAGGTCAACTTCAAGgtaagtttaaacatgattttactaactaactaatctgatttaaattttcagATCCGTCACAACATCGAGGACGGATCCGTCCAACTCGCCGACCACTACCAACAAAACACCCCAATCGGAGACGGACCAGTCCTCCTCCCAGACAACCACTACCTCTCCACCCAATCCGCCCTCTCCAAGGACCCAAACGAGAAGCGTGACCACATGGTCCTCCTCGAGTTCGTCACCGCCGCCGGAATCACCCACGGAATGGACGAGCTCTACAAGggaggtggaATGTTTCCaGAgcTcCAaACcCTtCAaTGGCCGATtATcAAGTAcAAGCAgcTcAGAGGAAGCGGTAATCGACAAGAGgttttaaaaatagatgaattctgtatcgaaaagttaaatttgcagGGCAAAGATATTCGCGTTGTTATGGAGGTGAATAGCAGGAAGCTGACGGTTATTCATGGAGTCGAGCCGATCGAGACCGTTTATTGTAACATGGAAGTTTCTAGGTATCCGAGTTTGAAgtgagtctttgttgtttgagaaattaaagtcaaattgcccggttttcagAATGAAGAACACAAATTTGTTCGTGATTGTAAACAACCAGGCTCAAGGTTTCCGATTAACTCTTCGTGGCGAAGATCGGGAGAATTTTCTGTCAACTGTCAGAAAATTTGCTTATATTTCGGAGACTCCAGTTAAGGATCATTTGAATCGCTCATCCACAAATACCGgtattctttattcttttaatagtttcaagtttc-3' | forward primer: 5'-TGCGATCCCAATATGTACAGCT-3' reverse primer: 5'-TACCGGTATTTGTGGATGAGCG-3' |
